# Hydrodynamic Delivery of Cre Protein to Lineage-Mark or Time-Stamp Mouse Hepatocytes *In situ*

**DOI:** 10.1371/journal.pone.0091219

**Published:** 2014-03-13

**Authors:** Katherine M. Sonsteng, Justin R. Prigge, Emily A. Talago, Ronald K. June, Edward E. Schmidt

**Affiliations:** 1 Department of Immunology and Infectious Diseases, Montana State University, Bozeman, Montana, United States of America; 2 Department of Mechanical and Industrial Engineering, Montana State University, Bozeman, Montana, United States of America; Southern Illinois University School of Medicine, United States of America

## Abstract

Cre-responsive fluorescent marker alleles are powerful tools for cell lineage tracing in mice; however their utility is limited by regulation of Cre activity. When targeting hepatocytes, hydrodynamic delivery of a Cre-expression plasmid can convert Cre-responsive alleles without inducing the intracellular or systemic antiviral responses often associated with viral-derived Cre-expression vectors. In this method, rapid high-volume intravenous inoculation induces hepatocyte-targeted uptake of extracellular molecules. Here we tested whether hydrodynamic delivery of Cre protein or Cre fused to the HIV-TAT cell-penetrating peptide could convert Cre-responsive reporters in hepatocytes of mice. Hydrodynamic delivery of 2 nmol of either Cre or TAT-Cre protein converted the reporter allele in 5 to 20% of hepatocytes. Neither protein gave detectable Cre activity in endothelia, non-liver organs, or non-hepatocyte cells in liver. Using mice homozygous for a Cre-responsive marker that directs red- (Cre-naïve) or green- (Cre-converted) fluorescent proteins to the nucleus, we assessed sub-saturation Cre-activity. One month after hydrodynamic inoculation with Cre protein, 58% of hepatocyte nuclei that were green were also red, indicating that less than half of the hepatocytes that had obtained enough Cre to convert one marker allele to green were able to convert all alleles. For comparison, one month after hydrodynamic delivery of a Cre-expression plasmid with a weak promoter, only 26% of the green nuclei were also red. Our results show that hydrodynamic delivery of Cre protein allows rapid allelic conversion in hepatocytes, but Cre-activity is sub-saturating so many cells will not convert multiple Cre-responsive alleles.

## Introduction

**Figure 1:**
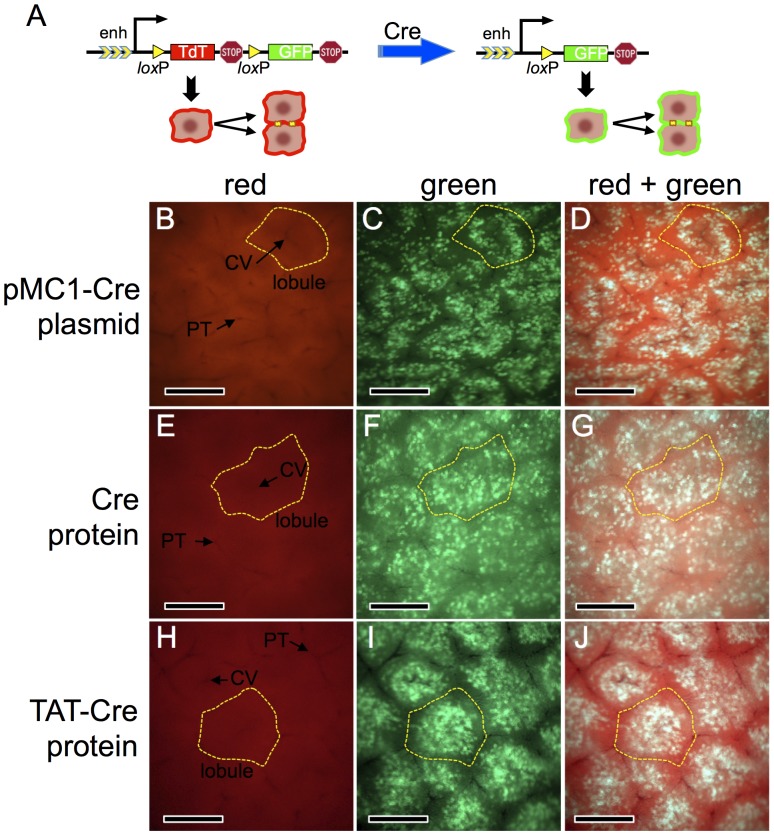
Cre activity in mouse hepatocytes following hydrodynamic delivery of Cre-expression plasmid or recombinant Cre proteins. Panel A, schematic of the *ROSA^mT−mG^* double-fluorescent reporter allele [2] used in this study. In the Cre-naïve state (left), the outer membranes of all cells fluorescent red. Following Cre exposure (right), the outer membrane of cells and all of their descendents fluoresce green. Panels B–J, young adult *ROSA^mT−mG/mT−mG^* mice were given hydrodynamic injections of lactated Ringer’s saline containing 25 µg of pMC1-Cre plasmid (panels B–D), 2 nmol of recombinant His-tagged Cre protein (panels E–G), or 2 nmol of recombinant His-tagged TAT-Cre protein (panels H–J). Mice were sacrificed ≥7 d later and whole livers were photographed using an epifluorescent dissecting steromicroscope. Panels B–D are the same frame photographed under different fluorescent channels, as are E–G and H–J. A representative lobule and representative central veins (CV) and portal triads (PT) are indicated. Scale bars = 0.5 millimeter.

Cre-dependent marker alleles have proven useful as cell lineage markers and as surrogate identifiers for cells having disruption of a Cre-dependent conditional allele [1]. The *ROSA26*-targeted dual-fluorescent reporter allele *ROSA^mT−mG^* (for *ROSA26*-membrane tdT-membrane EGFP) encodes a floxed membrane-targeted tdTomato (tdT) cistron followed by a membrane-targeted enhanced green fluorescent protein (EGFP) cistron (Fig. 1A) [2]. In mice bearing *ROSA^mT−mG^*, cells on Cre-naïve lineages exhibit strong red fluorescence in their outer membranes; cells on Cre-exposed lineages exhibit strong green membrane fluorescence [2]. Recently we developed a modified version of this allele, entitled *ROSA^nT−nG^* (for nuclear tdT-nuclear EGFP) that differs from *ROSA^mT−mG^* in that the red- and green-fluorescent proteins are localized to the nucleus instead of the outer membrane [3]. Because the color status of either *ROSA^mT−mG^* or *ROSA^nT−nG^* is passed to daughter cells, these alleles are useful tools for lineage tracing [2], [3]. However to be effective, these markers require precise control of Cre expression and use of a Cre system that does not disrupt the physiology being studied [4]–[7].

Stoichiometrically, Cre activity requires four molecules of the protein: a dimer at each of two appropriately positioned *lox*P sites [8]. However it is unknown how many more molecules than this are required for Cre to kinetically find a target-pair of *lox*P sites in the context of native chromatin structure within a nucleus and complete the reaction. The high incidence of “leaky expression” from otherwise inactive Cre alleles [4], [5], [7] suggests this number might be very low. Indeed, one of the greatest challenges in working with Cre-expressing transgenic mice has been the difficulty in preventing leaky Cre activity, leading to improper spatial or temporal allelic conversion [7]. In addition, some studies have shown that Cre, itself, can occasionally have “off-target” effects, likely through interactions with cryptic *lox*P-like sites in the genome [9], [10]. Ideally, one would like a system wherein Cre activity switched instantaneously from being truly “zero” to transiently attaining a modest level that could effectively recombine between *lox*P sites without causing off-target effects.

Hepatocytes are one of few cell types in adult mice that can proliferate and give rise to lineages in adult animals [3], [11], [12]. Several transgenic systems have been developed that could deliver Cre activity to *ROSA^mT−mG^* hepatocytes for lineage tracing; however most are problematic. For example, the inducible Cre transgenes we have tested [13], [14] exhibit varying degrees of leakiness in hepatocytes, which severely hinders lineage tracing. To achieve a finite window of Cre activity, initiating in a truly Cre-naïve animal and returning stably to “zero” Cre activity shortly thereafter, we sought a transient exogenous Cre-delivery system.

Transient delivery of exogenous Cre activity to hepatocytes can be performed using non-integrating virus-based vectors (e.g., [15], [16]). However, viral-derived vectors will express, in addition to Cre, pathogen-associated molecular pattern molecules (PAMPs) that can induce cellular antiviral responses and might alter the marked cells, themselves, or their interactions with the immune system [17]–[21]. To minimize such vector-induced artifacts, we began marking lineages by hydrodynamic delivery of a naked plasmid that would express only Cre protein. By this approach, mice are rapidly injected via the tail vein with a large volume of saline containing the plasmid [22], [23]. The ensuing hydrodynamic stress on the liver induces transient pores in hepatocytes, which allows entry of the plasmid [24]. Although there is some brief tissue damage associated with the procedure, hydrodynamic injections provide a means to introduce DNA into hepatocytes that does not show evidence of compromising hepatocyte longevity [25].

In ongoing efforts to further improve technologies for hepatocyte time-stamping or lineage-tracing, we tested whether marker alleles in hepatocytes could be converted *in situ* by hydrodynamic delivery of either recombinant Cre protein or Cre fused to the HIV-TAT cell-penetrating peptide (TAT-Cre) [26], [27]. We show that hydrodynamic delivery of Cre proteins can provide an effective means to synchronously deliver transient Cre activity at sub-saturating levels to hepatocytes.

## Materials and Methods

### Mouse Lines and Animal Care

This study was carried out in strict accordance with the recommendations in the Guide for the Care and Use of Laboratory Animals of the National Institutes of Health. All animal protocols were reviewed and approved by the Montana State University Institutional Animal Care and Use Committee. *Gt(ROSA)26Sor^tm4(ACTB−tdTomato, −EGFP)Luo^/J* [2] (abbreviated *ROSA^mT−^* ^mG^) mice were obtained from Jackson Laboratories (Stock # 007576) and were bred for production and maintenance in our colony. B6;129S6-Gt(ROSA)26Sor^tm1(ACTB−tdTomato*,−EGFP*)Ees^/J [3] (abbreviated *ROSA^nT−nG^*) mice were developed in our laboratory and are publicly available through Jackson Laboratories (Stock # 023035).

### Plasmids and Recombinant Proteins

pMC1-CRE plasmid contains a eukaryote-adapted Cre protein with a nuclear localization signal driven by a synthetic promoter consisting of the HSV-tk minimal promoter and polyomavirus enhancer [28]. His6-tagged Cre and TAT-Cre were expressed from PET28 vectors as described previously [26], [27], [29] and were purified on Ni-NTA columns. Proteins were eluted with imidazole by standard procedures, snap-frozen in liquid nitrogen, and stored at −80°C. Concentrations were typically 60 µM, and were diluted ∼ 100-fold into sterile lactated Ringer’s solution for injection.

### Hydrodynamic Injections

Hydrodynamic injections were performed as described previously [24], [25]. Briefly, the solute to be administered (plasmid or recombinant protein) was dissolved in a volume of lactated Ringer’s solution equaling 1/10^th^ the body-weight and this was rapidly injected into the tail vein (within 3–9 seconds) to impart hydrodynamic stress on the liver.

### AdCre Transduction

AdCre [15] grown, CsCl-purified, and titred by the Custom Services division at Vector Biolabs, was diluted into DMEM for retroorbital inoculations [30]. In a pilot experiment, log-dilutions from 10^8^ to 10^3^ PFU were tested and 2×10^6^ was found to closely approximate the number of cells converted by HDTV with 25 µg of pMC1. This was diluted into DMEM medium (50 µl) prior to injection.

### Fluorescent Analyses

Livers were observed whole at harvest using a Nikon SMZ-800 binocular dissecting microscope outfitted with an Epifluorescence package (Nikon) including an Exfo X-Cite 120Q UV light source, red (Nikon C3394 R/Dil 31002a) and green (Nikon C127265 EN/GFP 83457) filter sets, and a Nikon DS-Fi1 digital camera. For observing sections, samples were frozen in OCT medium (Tissue Tec). For sections prepared from *ROSA^mT−mG^* livers, 5-micron sections were cut on a cryomicrotome, lifted onto glass slides, fixed in 3∶1 acetone:ethanol, and mounted in Fluoromount-G with DAPI (Electron Microscopy Services). For *ROSA^nT−nG^* livers, samples were either frozen in OCT and sectioned as above, as described previously [3], or they were formalin pre-fixed first, as described in figure legends. In the later case, liver pieces were harvested into ice-cold buffered 10% formalin and incubated at 0–4°C 12–24 h with gentle agitation. Tissue samples were then incubated in 30% sucrose, 1X PBS with gentle agitation for 15 min, embedded in OCT, and frozen. After sectioning, pre-fixed sections were mounted directly without an on-slide fixation step. Images were taken on a Nikon Eclipse 80i microscope using a Nikon DS Ri1 digital camera, Nikon NIS Elements BR acquisition software, and standard red (TRITC), green (FITC), and DAPI filter sets.

### Data Analysis and Statistical Evaluation

For quantitative analyses of fluorescent signals, uniform settings were used for all image acquisition, monochromatic images for each filter set were captured independently, and digital fluorescent micrographs were analyzed using single-color channels within the “Histogram” function of Photoshop CS3 software, as described previously [31]. Numerical data was presented as mean±standard error of the mean and statistical significance was evaluated using a Student’s T-test with *P≤*0.05 considered significant. Graphs and figures were made in GraphPad Prism-6 or Microsoft PowerPoint.

## Results

### Hydrodynamic Delivery of Cre or TAT-Cre to Mice Can Induce Cre-dependent Recombination in Hepatocytes

In the absence of Cre activity, *ROSA^mT−mG/mT−mG^* or *ROSA^nT−nG/nT−nG^* mice exhibit no EGFP expression in any tissues including liver [2], [3], [15], [30]–[32]. Hydrodynamic delivery of 25 µg of pMC1-Cre plasmid, which uses a very weak ubiquitously active promoter [28] to drive Cre expression, into *ROSA^mT−mG/mT−mG^* mice resulted in ∼3%–10% of the hepatocytes stably converting to green, with preferential targeting of mid-zone hepatocytes (Fig. 1B–D, livers harvested >7d post hydrodynamic injection). Only hepatocytes exhibited allelic conversion in liver (Fig. 1B–D), and surveys of the animals showed no evidence of Cre activity elsewhere (i.e., no green cells; data not shown).

**Figure 2:**
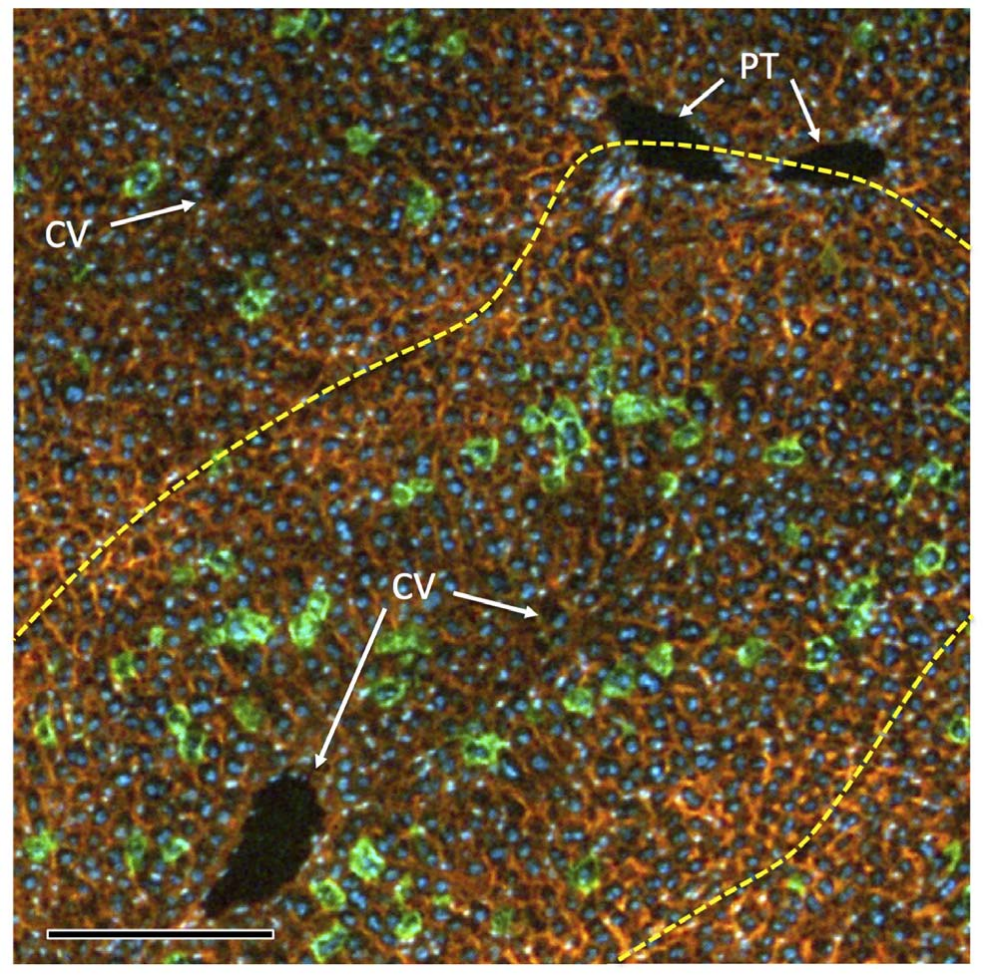
Distribution of marked hepatocytes in a liver lobule. A *ROSA^mT−mG/mT−mG^* mouse received hydrodynamic delivery of 2 nmol of His-tagged TAT-Cre protein, was harvested 7 d later, and DAPI-stained cryosections of the liver were photographed. Yellow dotted line circumscribes a lobule. Representative central veins and portal triads are indicated. Note that green hepatocytes predominate in the mid-zonal region, and are rare in the outer region of lobules. Scale bar = 50 µmeter.

To test whether conversion of hepatocytes in *ROSA^mT−mG/mT−mG^* mice could also be achieved using hydrodynamic delivery of purified recombinant Cre protein, we inoculated 2 nmol of either Cre or TAT-Cre protein, and harvested mice ≥7 days later. Either protein converted ∼5%–20% of the hepatocytes (Fig. 1 panels E–J). Like using pMC1-Cre plasmid, the protein inefficiently converted hepatocytes near the lobular periphery and most efficiently converted mid-zone hepatocytes (Fig. 2); however using the recombinant Cre proteins, we observed more effective conversion of centrolobular hepatocytes than that achieved with plasmid (compare Fig. 1 D to 1G & J).

### Neither Cre Nor TAT-Cre Cause Recombination in Non-liver Organs or in Non-hepatocyte Cell Types in the Liver

**Figure 3:**
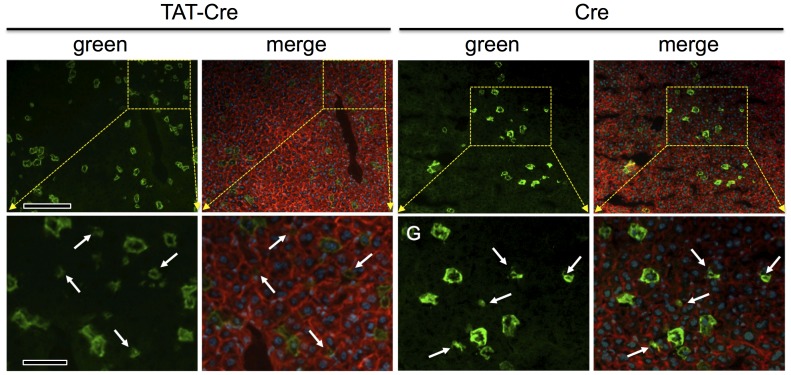
Hydrodynamic delivery of Cre or TAT-Cre protein targets hepatocytes. Cryosections of livers harvested from mice as in Fig. 1 were photographed as in Fig. 2. The yellow dotted boxes in upper panels correspond to the enlarged images in lower panels, as indicated. Regions containing regions of green membrane that appeared smaller than hepatocytes are shown (white arrows) The merged images revealed that these small regions of green membrane generally did not contain within them a DAPI-stained (blue) nucleus suggesting these were not small non-hepatocyte cells, but rather, are the “tips” or “edges” of large hepatocytes whose bulk lay in adjacent sections, as further evaluated in Fig. 4. Scale bars: in upper panels  = 50 µmeter; in lower panels  = 16 µmeter.

**Figure 4:**
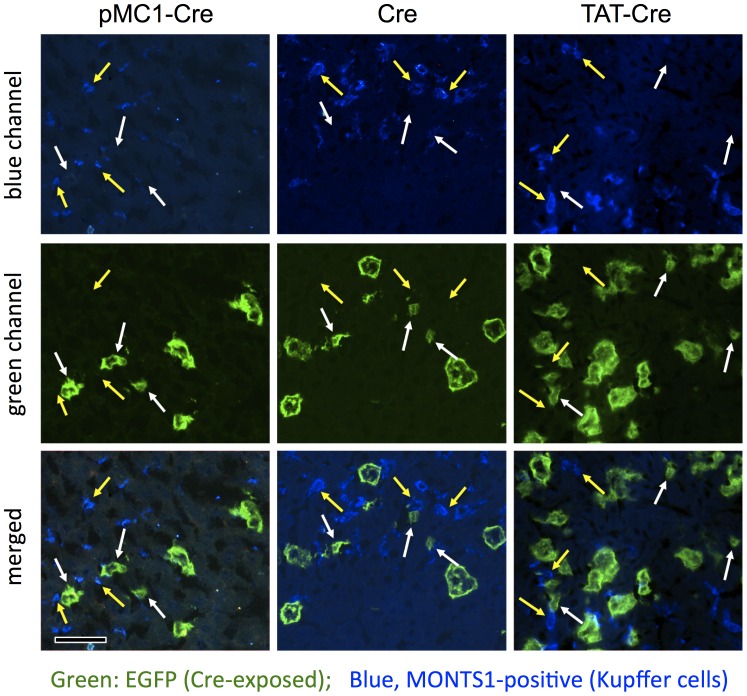
No evidence of Cre activity in MONTS1-positive Kupffer cells following hydrodynamic inoculation with pMC1-Cre, Cre protein, or TAT-Cre protein. One week after hydrodynamic inoculations in *ROSA^mT−mG/mT−mG^* mice, livers were harvested and cryosections were stained for MONTS1 (blue) to examine frequencies of co-localization of green membrane with MONTS1 staining (i.e., Cre-converted Kupffer cells). At least 300 green cells were evaluated for each condition and none were identified as MONTS1-positive. Images show MONTS1 staining only (top panels), Cre conversion only (middle) or merged images (bottom). White arrows denote representative regions of green membrane that appear too small to be hepatocytes, as in Fig. 3. Yellow arrows denote Kupffer cells that appeared to roughly co-localize with green membranes. In each case, the green membranes were not MONTS positive and the MONTS positive Kupffer cells were adjacent to green cells, but not themselves green. Scale bars 20 µmeters.

In liver of *ROSA^mT−mG/mT−mG^* mice that received hydrodynamic injection with pMC1-Cre plasmid, Cre protein, or TAT-Cre protein, all endothelial cells remained red (Figs. 1–3). Also, whole body surveys of the mice using the epifluorescent dissecting microscope showed no green cells along the vasculature or within organs other than liver (organs examined included skin, body-wall, lung, heart, thymus, kidney, spleen, large- and small-intestine, diaphragm, pancreas, bladder, testis, uterus, and brain; data not shown). Thus, none of these conditions showed evidence of inducing Cre activity in endothelial cells or in any non-liver organs, except that we occasionally observed some green fluorescence near the site of injection in the tail when using TAT-Cre protein (data not shown). Using hydrodynamic injection of pMC1-Cre, Cre, or TAT-Cre, the predominant converted cells in liver were hepatocytes; although on cryosections some smaller regions of green membrane were observed that were either not hepatocyte membranes or were membranes of hepatocytes that were predominantly out of the plane of section (Fig. 3, arrows). To distinguish these possibilities, especially since TAT-Cre has been shown to induce Cre activity in some cultured cell types [26], [29], cryosections of *ROSA^mT−mG/mT−mG^* liver that had been hydrodynamically exposed to Cre, TAT-Cre, or pMC1-Cre were stained for the macrophage/Kupffer cell marker MONTS1 (Fig. 4) [3], [33]. Using MONTS1 staining on cryosections, we assessed ∼300 arbitrary green cells (Cre-converted) each on liver sections from mice hydrodynamically inoculated with either pMC1-Cre plasmid, Cre protein, or TAT-Cre protein, and we were unable to identify any cells in any conditions in which membranes were both MONTS1-positive (blue, e.g., yellow arrows in Fig. 4) and EGFP-positive (Cre-exposed, e.g., white arrows in Fig. 4). In all cases we examined, the small regions of green membrane were not MONTS1-positive and the MONTS1-positive areas near green membranes did not co-localize, but were adjacent or nearby cells. Whereas we cannot exclude the possibility that hydrodynamic inoculation with pMC1-Cre, Cre protein, or TAT-Cre protein could occasionally target non-hepatocyte cell types, under the conditions we used, such events were not detected.

### Levels of Inflammation Following Hydrodynamic Delivery of pMC1-Cre Plasmid or Cre Protein

**Figure 5:**
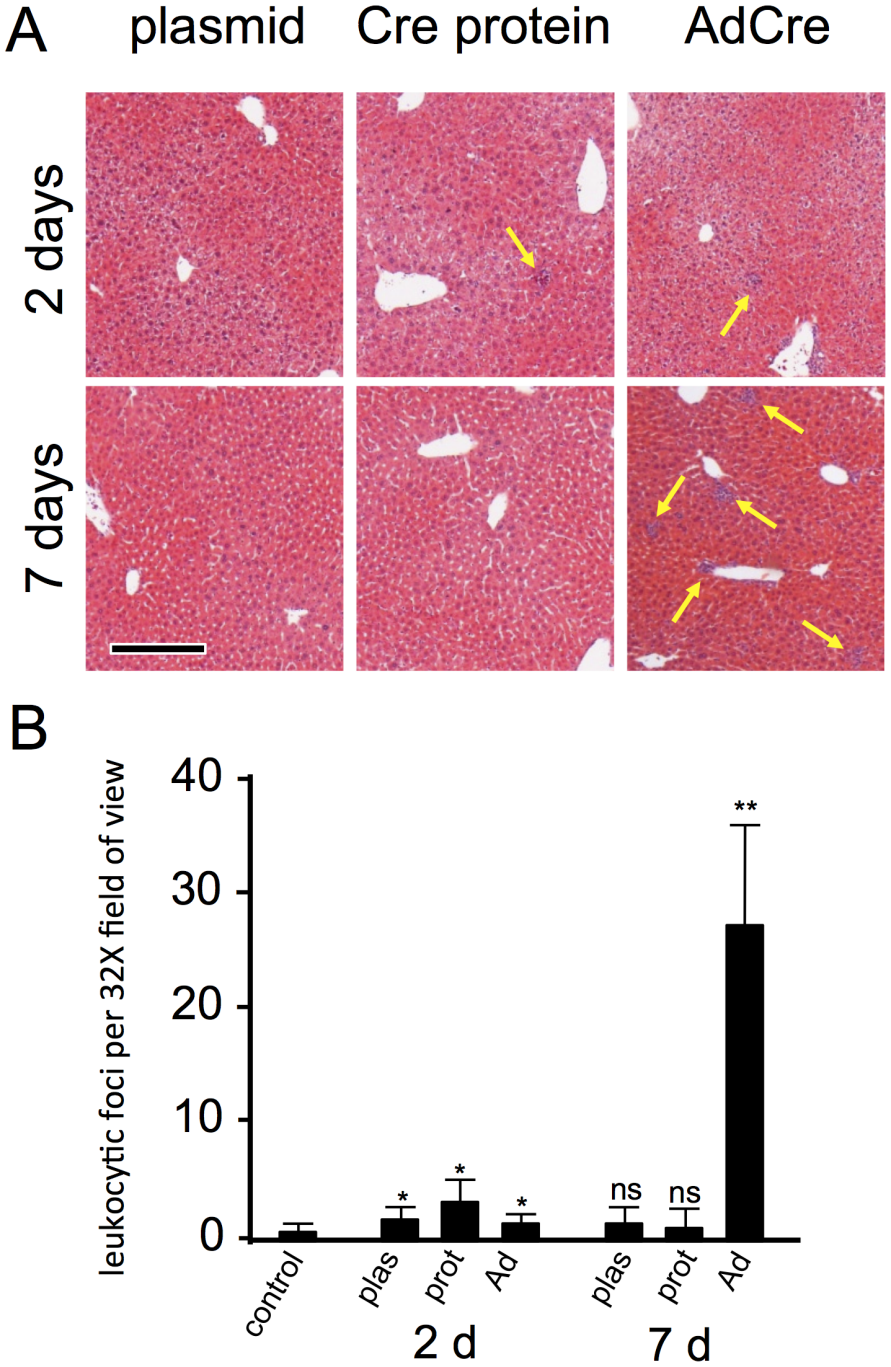
Hepatic inflammation following hydrodynamic delivery of pMC1-Cre or Cre protein, or non-hydrodynamic delivery of AdCre vector. Mice were uninoculated (control), were inoculated hydrodynamically with 25 µg of pMC1-Cre protein or 2 nmol of Cre protein, or they received 2×10^6^ PFU of CsCl-purified AdCre vector retroorbitally, as indicated. Mice were harvested 2 or 7 days later and H&E paraffin sections were evaluated for the number of leukocytic foci per field of view at 32× magnification (av. 8.5×10^3^ hepatocytes/field). Representative foci are indicated by yellow arrows in panel A. Scale bar 100 µmeters. Note, frames in panel A were taken at 100× to better reveal the leukocytic foci, and therefore contain only (3.2) *^−^* ^2^, or ∼ 9%, as many cells or leukocytic foci as those quantified in paned B. Quantitative data are presented in panel B. *, *P≤*0.05 compared to control; **, *P≤*0.05 compared to control; ns, not significantly different than control. The three conditions did not differ significantly from each other at 2 d.

To compare levels of hepatic inflammation induced by hydrodynamic delivery methods with those arising from use of a virus-derived vector, we analyzed livers harvested 2- or 7-days after hydrodynamic delivery of 25 µg pMC1-Cre, hydrodynamic delivery of 2 nmol Cre protein, or transduction with 2×10^6^ PFU of CsCl-purified AdCre vector (Fig. 5A). These conditions were determined in a pilot study to convert a similar proportion of hepatocytes (data not shown). In unchallenged livers, almost no leukocytic foci could be found. At 2 d after Cre exposure, all three conditions resulted in small increases in leukocytic foci but no significant differences between any of the Cre exposures were measured (Fig. 5B). However, at 7 d, whereas livers that received either hydrodynamic delivery of pMC1-Cre or Cre protein showed no significant difference in leukocytic foci as compared to each other or to untreated controls, the livers that were exposed to AdCre exhibited >20-fold more leukocytic foci (Figs. 5A & B).

### Conversion Frequencies of Multiple Target Alleles within Hepatocytes Following Hydrodynamic Delivery of pMC1-Cre or Cre Protein

Most if not all previous studies of Cre activity in mice used systems in which Cre protein was synthesized by the target cells. This results in amplification of Cre protein levels by translation and, in most cases, transcription. Thus, a single copy of a gene can be transcribed multiple times (this number dependent on the transcriptional activity of that gene), and each mRNA issued can be translated multiple times (this number dependent on the translational efficiency and stability of that mRNA). Indeed, the suspected reason that “leaky” Cre activity can be such a problem in some transgenic mouse models is that even expression of a single functional mRNA in a cell might, through multiple rounds of translation, issue enough copies of Cre protein to recombine a Cre-dependent allele. However, by delivering Cre protein directly to hepatocytes in the current study, amplification was prevented. To examine whether this resulted in increased levels of incomplete allelic conversion we used a different dual-fluorescent marker, *ROSA^nT−nG^*, from which the expressed tdT and EGFP proteins are targeted to the nucleus [3]. Unlike outer membranes, which will contact membranes of adjacent cells, nuclei are distinctly separated from those of adjacent cells. This makes it easier to evaluate allelic properties of individual cells using *ROSA^nT−nG^*. We also used homozygous mice (*ROSA^nT−nG/nT−nG^*), such that diploid cells would have two Cre-dependent alleles that could be independently evaluated based on the fluorescence of the cell’s nuclei. Moreover, adult mouse hepatocytes are frequently polyploid, further increasing the number of Cre-dependent alleles that a cell might possess. Following Cre exposure, if sufficient time were allowed for turnover of pre-formed fluorescent proteins, the red-versus-green fluorescence of nuclei should be proportional to the ratio of non-recombined to Cre-recombined marker alleles within that cell.

**Figure 6:**
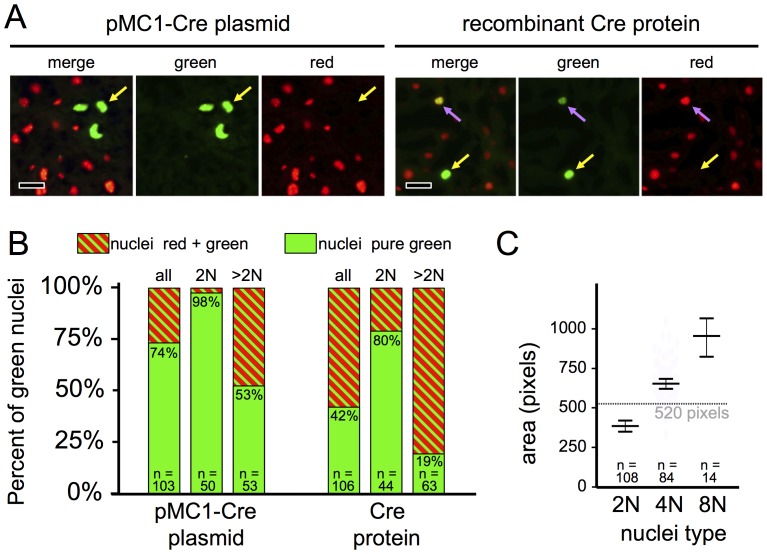
Sub-saturating Cre activity following hydrodynamic inoculation with pMC1-Cre or Cre protein. *ROSA^nT−nG/nT−nG^* mice received hydrodynamic inoculation with 25 µg of pMC1-Cre or 2 nmol of Cre protein, as indicated, and were harvested 1 month later. A, representative fluoromicrograph images of cryosections. Yellow arrows denote nuclei that are green and not red, denoting conversion of all *ROSA^nT−nG^* alleles in that cell. Lavender arrows denote nuclei that are stably red and green, indicating that one or more *ROSA^nT−nG^* alleles in that cell converted to green, and one or more alleles did not. Scale bars 10 µmeters. B, Quantification of the ratios of green only to green+red alleles in mouse livers one month after hydrodynamic delivery of either pMC1-Cre plasmid or Cre proteins. See panel C for description of how we discriminated between “apparently 2N” from “>2N” hepatocytes. C, Morphometric analysis of nuclei areas in cryosections. The areas of 300 nuclei were determined by pixel-counts on Photoshop CS3 software and segregated into 2N, 4N, and 8N categories (16N nuclei were excluded from this figure). Graphs show the mean±the 95% confidence interval, as determined in GraphPad Prism-6 software. Based on this, an area cutoff of 520 pixels (dotted line) was chosen to separate “apparently 2N” from >2N nuclei. Apparently 2N hepatocytes appeared mononucleate in the plane-of-section and that nucleus was apparently 2N. Because higher-ploid nuclei that are cut off-center will appear 2N and some bi-nucleate cells will only have one nucleus in the plane of section, the “apparently 2N” group will contain an unknown percentage of higher-ploid cells.

pMC1-Cre plasmid or Cre protein were hydrodynamically inoculated into *ROSA^nT−nG/nT−nG^* mice. Livers were harvested 30 d later and cryosections were evaluated by fluorescence microscopy (Fig. 6A). Using pMC1-Cre, 26% of the hepatocyte nuclei that exhibited green fluorescence also had red fluorescence (Fig. 6B). This indicated that, despite transcription- and translation-driven amplification, roughly a quarter of the cells that exhibited any Cre activity accumulated only sub-saturating levels of Cre (see Discussion). With Cre protein, 58% of the nuclei that exhibited green fluorescence also had red fluorescence (Fig. 6B), indicating that most cells that took up enough Cre protein to convert one allele did not covert all alleles in that cell. To determine whether the ploidy of individual hepatocytes influenced the ability of Cre to convert all alleles, we evaluated the sizes of nuclei and segregated these into apparent 2N, 4N, and 8N groups (Fig. 6C) [3]. Some uncertainty arises from large nuclei that were sectioned off-center, giving rise to smaller areas; however the data yielded good segregation of the nuclei classes and allowed us to set a threshold of <520 pixels as being “apparently 2N”, and >520 pixels being >2N (Fig. 6C). We then separately analyzed green “apparently 2N” hepatocytes (appearing, within the plane-of-section, to have a single nucleus of 2N area) and >2N (bi-nucleate within the plane of section or having a nuclear area >520 pixels). In livers that received pMC1-Cre, 98% of the green nuclei in apparently 2N hepatoctyes were purely green, whereas only 53% of the green nuclei in higher-ploid hepatocytes were purely green (Fig. 6A). Using Cre protein, these values were 80% complete conversion in 2N, and only 19% in higher-ploid, hepatocytes (Fig. 6B; see Discussion).

### Kinetics of Allelic Conversion Following Hydrodynamic Delivery of pMC1-Cre or Cre

**Figure 7:**
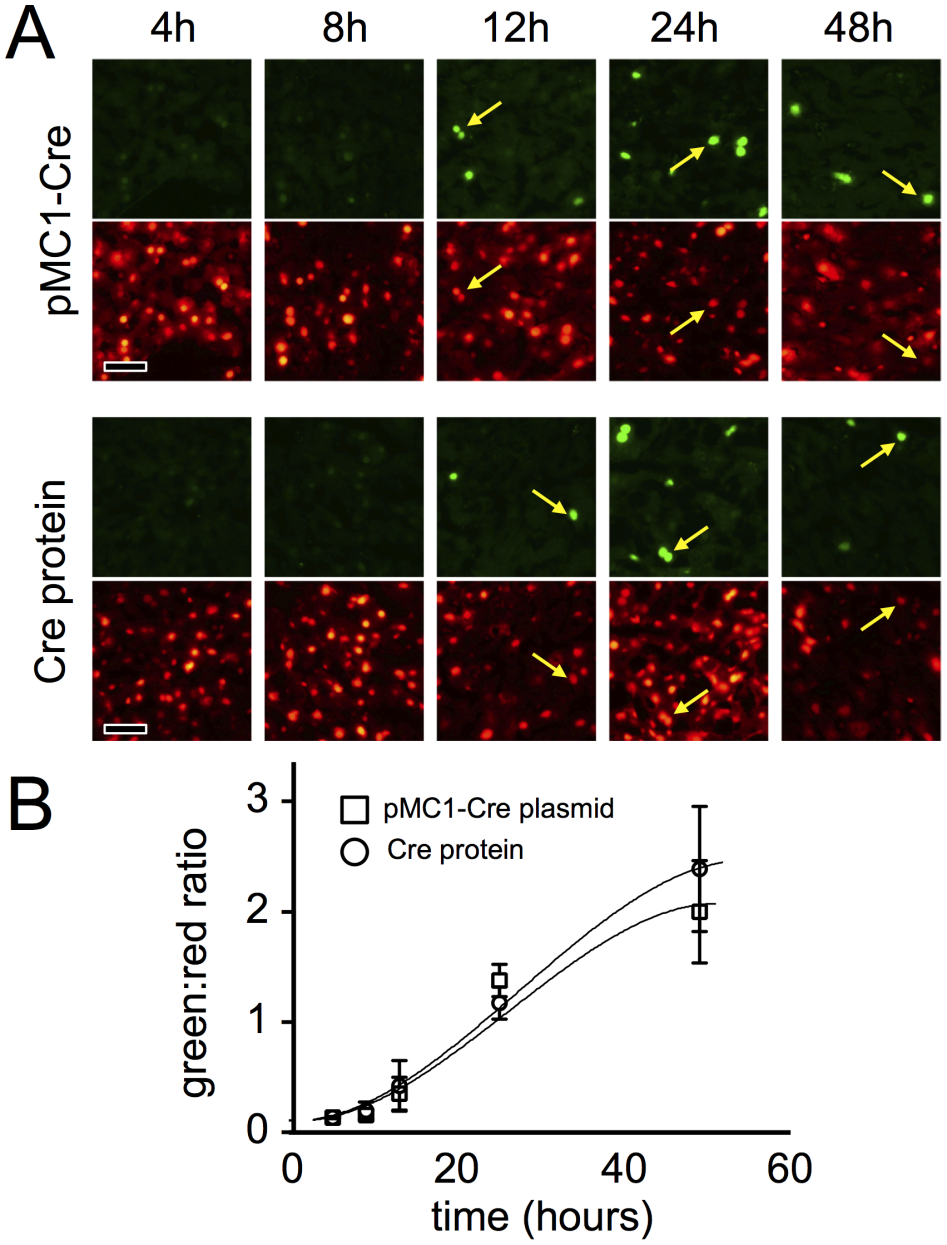
Kinetics of *ROSAnT-nG* conversion in hepatocytes following hydrodynamic delivery of pMC1-Cre plasmid or Cre protein. Groups of *ROSAnT-nG/nT-nG* mice were inoculated hydrodynamically with 25 µg pMC1-Cre plasmid or 2 nmol Cre protein and were harvested at the indicated times thereafter. Thirty apparently diploid nuclei were analyzed on cryosections of livers from each mouse. A, representative photomicrographs. Yellow arrows indicate the same nuclei in green and red panels to ease comparisons. Scale bars  = 25 µmeter. B, cumulative data. Approximate best-fit lines were drawn by hand.

Since pMC1-Cre plasmid needs to be transcribed and its mRNA translated in the target cells, we examined whether hydrodynamic delivery of Cre protein induced more rapid conversion of a double-fluorescent reporter than did delivery of pMC1-Cre. Sets of *ROSA^nT−nG/nT−nG^* mice were given hydrodynamic injections with either 25 µg of pMC1-Cre or 2 nmol of Cre protein and were harvested over a time-course thereafter. In both cases, nuclear green fluorescence was first detected between 8 and 12 h after inoculation (Fig. 7). No substantial difference was measured in the kinetics of nuclear fluorescence conversion between the two groups of mice (Fig. 7B), suggesting that the anticipated “delay” for transcriptional and translational processes using the plasmid was either immeasurably brief or was matched by other delays occurring when Cre protein is delivered directly.

## Discussion

Cre-*lox*P technology has become an important tool for *in situ* genome manipulation in mouse models. Ongoing improvements or modifications of this technology continue to augment its utility. In the study presented here, we explored the use of hydrodynamic delivery of Cre protein for conversion of Cre-dependent marker alleles in hepatocytes. We show that this approach can provide effective hepatocyte cell-lineage marking. Forty-eight hours after inoculation, the levels of hepatic inflammation induced by standard delivery of AdCre, hydrodynamic delivery of pMC1-Cre, or hydrodynamic delivery of Cre protein were all similarly low. At 7 d post-inoculation, however, AdCre-exposed livers showed dramatically increased levels of inflammation whereas both hydrodynamic exposures had returned to baseline levels. The delayed inflammation in the AdCre-exposed liver is consistent with this being a response to expression and presentation of vector-encoded PAMPs by the transduced cells. These results verified that hydrodynamic delivery of purified DNA or protein induced less inflammation than did use of an AdCre vector, which represents an important advantage of the hydrodynamic systems.

### Hydrodynamic Delivery of Cre Protein Versus pMC1-Cre Plasmid to Hepatocytes

Hydrodynamic delivery has been shown to induce hepatocytes to internalize plasmids, proteins, RNAs, and other molecules [25], [34]–[37]. However, to our knowledge, it had not previously been shown that hydrodynamic delivery of Cre protein could yield effective recombination of Cre-dependent alleles in liver. The Cre-*lox*P reaction requires coincident assembly of two pair of Cre proteins - one pair on each of two appropriately positioned *lox*P sites. The probability of the six components of this reaction (4 molecules of Cre and 2 *lox*P sites) coincidentally assembling might be expected to be quite low in the context of normal chromatin structure within a mammalian nucleus unless levels of Cre protein were in large excess, such that this could drive the reaction. We show that hydrodynamic inoculation with either 2 nmol of Cre protein or 25 µg of pMC1-Cre plasmid resulted in similar proportions of hepatocytes undergoing at least one Cre-dependent allelic conversion. pMC1-Cre is 4545 bp in size, so the 25 µg inoculum is ∼ 8 pmol, or 1/250^th^ of the concentration of Cre protein we were inoculating. Assuming both the protein and the plasmid preparations contained similar proportions of active to inactive molecules, the 250-fold higher concentration of Cre protein could compensate for much of the amplification that might occur through transcription and translation of pMC1-Cre plasmid.

It is interesting that, whereas hydrodynamic delivery of pMC1-Cre yields a strong targeting-bias toward mid-zone hepatocytes, Cre protein also effectively converted centrolobular hepatocytes (Fig. 1). Cre protein, a globular molecule of MW ∼3×10^4^ Da, will have a Stoke’s radius many times smaller than that of pMC1-Cre plasmid, a distended molecule of MW ∼3×10^6^ Da. Thus, larger pores might be required to allow entry of pMC1-Cre into hepatocytes. The architecture of the liver lobule might affect the size of pores that are induced in hepatocytes of each zone during hydrodynamic stress. Further studies will be required to determine how hydrodynamic challenges impact hepatocytes, what roles lobular architecture play on this, and how the size, shape, surface charge, and diffusion characteristics of molecules delivered by hydrodynamic injection might be differentially affected by these parameters.

Cre protein, upon entering hepatocytes, only needed to localize to the target *lox*P pair and assemble with 3 other Cre proteins to execute allelic conversion. By contrast, pMC1-Cre plasmid, upon entry, required nuclear translocation and transcription of the plasmid; maturation and nuclear export of the mRNA; and then translation and nuclear localization of the active Cre protein for activity. Thus, we expected that hydrodynamic injection with Cre protein would induce more rapid red-green conversion than would pMC1-Cre. Instead, both Cre and pMC1-Cre induced similarly red-green conversion (Fig. 7), indicating that the process occurred at a similar rapid rate in both cases. This suggests that either the anticipated delays for expression of pMC1 plasmid were negligible within the parameters of the system used or that, following hydrodynamic delivery into hepatocytes, the “route” or process by which Cre protein transits to the nuclear *lox*P sites is slow, thus causing a delay that approximates the time required for expression of pMC1-Cre. Since the first step with either Cre protein or plasmid is transit to the nucleus, this might suggest different mechanisms of nuclear localization are at play.

### Activity of the TAT Cell-penetrating Peptide in Hydrodynamic Delivery of Proteins

The TAT cell-penetrating peptide has been used to direct diverse molecules into cells [38]. Here we compared hydrodynamic delivery of either recombinant Cre or TAT-Cre for converting Cre-dependent markers in mice. Importantly, the TAT-Cre protein we used was previously shown to function in cell culture systems [26]. Nevertheless, we did not find any substantial difference in the distribution of cells that exhibited conversion of a Cre-dependent marker in liver following hydrodynamic delivery of Cre versus TAT-Cre. In particular, TAT-Cre did not convert Cre-dependent alleles in the vasculature or in Kupffer cells (Figs. 1–4). In whole animal surveys, the only difference we were able to find between the two proteins was that, occasionally, we found evidence of green fluorescence near the injection site in the tails of animals that received hydrodynamic injections of TAT-Cre (data not shown). The lack of wide-spread Cre activity when using TAT-Cre might be related to previous reports of charged molecules in the serum interfering with activity of the cell-penetrating peptide [39]. Alternatively, Kupffer and endothelial cells might be refractory to the TAT cell-penetrating peptide. Further studies will be required to understand the behavior of this peptide in animals.

### Saturating Versus Sub-threshold Cre Activity

The reason that Cre-dependent reporter alleles such as *ROSA26* [1], *ROSA^floxSTOP−YFP^* [40], or *ROSA^mT−mG^* [2] have proven useful as surrogate markers for cells in which other Cre-dependent alleles have been recombined is that, typically, any cell expressing an active Cre protein reliably exhibits recombination of all Cre-responsive alleles. By using *ROSA^nT−nG/nT−nG^* mice [3], we were able to assess the ability of Cre exposure conditions to recombine multiple Cre-dependent alleles in a given cell. Thus, cells in these mice will have two independently recombining alleles per diploid genome, and because adult mouse hepatocytes are commonly 2N, 4N, 8N, or 16N, with an average nuclear ploidy of ∼3.4N [3], different cells will vary from having 2 to 16 independently recombining *ROSA^nT−nG^* alleles. Using hydrodynamic delivery of pMC1-Cre, 98% of the “apparently 2N” hepatocytes that converted one *ROSA^nT−nG^* allele converted both alleles, indicating that Cre protein accumulated to near-saturating levels for recombining two Cre-dependent alleles in these cells. However, only 53% of higher-ploid hepatocytes that showed conversion of at least one *ROSA^nT−nG^* allele (detected as green nuclear fluorescence) showed conversion of all *ROSA^nT−nG^* alleles (detected as absence of red nuclear fluorescence), suggesting that these same conditions yielded sub-threshold levels of Cre protein for conversion of four or more alleles. Using Cre protein, these values were considerably lower, with a small fraction of the higher-ploid cells (19%) showing complete conversion. These observations impact both the utility of the system and our understanding of Cre-dependent recombination in cells.

For time-stamping or lineage-tracing analyses, converting one allele is sufficient – the cell and its descendants will be permanently marked. Indeed, this might be a preferable situation, as having sub-saturating Cre activity should diminish the likelihood of Cre causing collateral damage to the genome through interactions with cryptic endogenous *lox*P-like sites. On the other hand, for studies where a marker allele is used as a “surrogate identifier” of cells in which a second Cre-dependent “conditional-functional” allele has been Cre-exposed, sub-saturating Cre activity would be undesirable. Many “marked cells” (Cre-exposed) would have not recombined the conditional-functional allele, and many unmarked cells (appearing Cre-naïve) might have recombined the conditional-functional allele. In this context, it is noteworthy that, since hydrodynamic delivery of a protein provides a non-amplifying Cre-exposure, the problem of sub-saturating Cre activity would be very difficult to overcome. At best, incremental increases in activity might come from delivering more protein or increasing the *in vivo* stability of the protein. With plasmid, however, the problem should be easily surmounted by simply increasing the strength of the promoter. In this context, it is noteworthy that the MC1 promoter was specifically designed to give very weak ubiquitous expression [28]. In future studies, it will be useful to test plasmids in which Cre expression is driven by promoters spanning a wide range of activities [41] to determine which might be best for use in hydrodynamic delivery of plasmid-encoded Cre to reliably convert a conditional-functional allele and a surrogate marker allele.

### Comparison of Hepatocyte Cre-exposure Systems: which is Best Suited to a Given Situation?

If one wishes to convert a Cre-dependent allele in all hepatocytes initiating at hepatocyte differentiation, likely the best approach is to breed that allele across the *AlbCre* transgene [42]. *AlbCre* is induced coincident with the differentiation of hepatocytes from progenitor cells, such that even in fetal stages, all hepatocytes very rapidly convert Cre-responsive reporters [32]. However, if one wants to delay Cre activity until later times or restrict it to a marked subset of hepatocytes, *AlbCre* is not useful. Inducible Cre transgenes can be used for this, but these will often show leakiness (see Introduction). Exogenous delivery systems, which can be either a virus-derived vector or hydrodynamic delivery, are best able to prevent leakiness. Virus-derived vectors provide reliable synchronous delivery of Cre activity to hepatocytes [31], and by titrating the dose of vector delivered, one can convert anywhere from a few to perhaps all hepatocytes [16], [31]. However, as shown here (Fig. 5), this can induce inflammation. Hydrodynamic approaches also allow synchronous leak-proof induction of Cre in hepatocytes. The primary advantage of hydrodynamic delivery is the diminished level of inflammation associated with this approach (Fig. 5). The primary disadvantage is that, typically, only a zonally-biased subset of hepatocytes are marked (Figs. 1&2). Finally, the predominant difference we note between hydrodynamic delivery of a Cre-expression plasmid versus Cre protein is that the plasmid can more effectively convert multiple alleles in a single cell whereas the protein shows less zonal bias in the converted hepatocytes (Fig. 1). In summary, each Cre-delivery system has advantages and disadvantages for any given situation. The investigator needs to choose the system that best suits the situation at hand.
